# BARIATRIC SURGERY IMPACT ON GASTROESOPHAGEAL REFLUX AND DENTAL WEAR: A SYSTEMATIC REVIEW

**DOI:** 10.1590/0102-672020190001e1466

**Published:** 2019-12-20

**Authors:** Ana Virgínia Santana Sampaio CASTILHO, Gerson Aparecido FORATORI-JUNIOR, Silvia Helena de Carvalho SALES-PERES

**Affiliations:** 1 Department of Pediatric Dentistry, Orthodontics and Public Health, Bauru School of Dentistry, University of São Paulo, Bauru, SP, Brazil.

**Keywords:** Bariatric surgery, Gastroesophageal reflux, Tooth wear, Tooth erosion, Cirurgia bariátrica, Refluxo gastroesofágico, Desgaste Dentário, Erosão dentária

## Abstract

**Introduction:**

Several oral problems may be perceived in individuals who were submitted to bariatric surgery, due to metabolic and behavioral changes relative to diet and oral hygiene. Tooth wear appears to suffer impact after bariatric surgery, because there may be an increase in gastroesophageal reflux.

**Objective:**

To systematically review the literature regarding the impact of bariatric surgery on gastroesophageal reflux and tooth wear.

**Method:**

The following databases were accessed by two independent, calibrated examiners: PubMed, Medline, Lilacs, Scielo and Cochrane using the following descriptors: “bariatric surgery” AND “dental erosion” OR “bariatric surgery” AND “dental erosion” AND “gastroesophageal reflux disease”. After excluding duplicate studies, 12 studies were initially evaluated by the title and abstract. The excluded studies were those without relevance to the present research, literature review studies and case reports. Thus, four articles were included in this study. All the articles evaluated indicated high association between gastroesophageal reflux and tooth wear in patients submitted to bariatric surgery. Association of these outcomes was more evident six months after the surgical procedure.

**Conclusion:**

Patients submitted to bariatric surgery showed higher prevalence of gastroesophageal reflux and tooth wear.

## RESUMO

**Introdução:**

Diversos problemas bucais podem ser percebidos nos indivíduos que foram submetidos à cirurgia bariátrica, frente às mudanças metabólicas e comportamentais referentes à alimentação e higienização. O desgaste dentário parece sofrer impacto após a cirurgia bariátrica, uma vez que pode ocorrer o aumento do refluxo gastresofágico após a operação.

**Objetivo:**

Analisar o impacto da cirurgia bariátrica no refluxo gastresofágico e no desgaste dentário por meio de uma revisão sistemática da literatura.

**Método:**

As seguintes bases de dados foram acessadas por dois examinadores independentes e calibrados: PubMed, Medline, Lilacs, Scielo e Cochrane usando os descritores: “bariatric surgery” AND “dental erosion” OR “bariatric surgery” AND “dental erosion” AND “gastroesophageal reflux disease”. Após a exclusão dos estudos duplicados, 12 estudos foram avaliados inicialmente pelo título e resumo. Foram excluídos os sem relevância para a presente pesquisa, os de revisão da literatura e os relatos de caso. Sendo assim, foram incluídos neste estudo quatro artigos. Todos os artigos avaliados indicaram uma alta associação entre refluxo gastresofágico e desgaste dentário em pacientes submetidos à cirurgia bariátrica. A associação destes desfechos foi mais evidente após seis meses do procedimento cirúrgico.

**Conclusão:**

Pacientes submetidos à cirurgia bariátrica mostram maior prevalência de refluxo gastresofágico e desgaste dentário.

## INTRODUCTION

Obesity is a chronic inflammatory disease that may be related to different comorbidities such as diabetes mellitus type 2, arterial hypertension, dyslipidemia, atherosclerosis, arthritis, obstructive sleep apnea syndrome, gastroesophageal reflux, and endocrine disorders, among others^7^.

Obesity surgery is the most effective way to lose weight in the long term, since it promotes reduction in the volume of the stomach and consequently, reduced the ingestion of food^3^. Bariatric surgery has been related to improvement in the systemic conditions and to aggravation of oral conditions, specifically to the increase in gingivitis^22^ and periodontitis^19^ tooth wear^17^ and dental caries^15^. This occurs due to the changes in metabolic patterns as a result of the changes in dietary and oral hygiene habits. 

Among the oral problems that affect obese individuals submitted to bariatric surgery, tooth wear appears to suffer impact after surgical treatment, since an increase in gastroesophageal reflux may occur after the surgery^1^. According to Litonjua et al.^9^, tooth wear is a multifactorial condition caused by the interaction among chemical, biological and behavioral factors, and results in dental hard tissue loss without the involvement of a carious process, due to the processes of erosion, attrition, abrasion and abfraction. Tooth wear by erosion is the loss of dental structure due to the presence of acids, which may be of intrinsic origin, such as gastroesophageal reflux, or extrinsic origin such as those related to diet, medication, lifestyle and environmental factors^5^ ^,^ ^10^. Dental erosion generally occurs in conjunction with abrasive processes, increasing the severity of tooth wear^23^.

Obese individuals with or without comorbidities are more exposed to the prevalence of tooth wear which, in its progression, leads to dentin hypersensitivity. Surgical treatment of obesity appears to be related to the prior clinical condition of the patient; however, some studies have shown that after bariatric surgery, patients may present with gastroesophageal reflux^6^ ^,^ ^13^. In view of the foregoing, it is relevant to understand how tooth wear progresses in the presence of gastroesophageal reflux in the individual submitted to bariatric surgery, because information about this topic continues to be inconclusive in the literature^20^. 

Considering that patients submitted to bariatric surgery present a high rate of gastroesophageal reflux and that this may be associated with tooth wear, the aim of this study was to evaluate the occurrence of tooth wear after bariatric surgery by means of a systematic review of the literature.

## METHODS

This study followed the rules proposed by PRISMA^8^. For this systematic review of the literature, PICO (Population, Intervention, Comparison and Results) was adopted to answer the following question: “What impact does bariatric surgery have on gastroesophageal reflux and tooth wear in patients who were submitted to bariatric surgery?”.

For population, studies of patients who were submitted to bariatric surgery were included, without restriction on the surgical technique used. 

### Eligibility of articles

The inclusion criteria for this systematic review were: cross-sectional and longitudinal clinical studies. The exclusion criteria for the systematic review were: reviews of the literature and case studies. No restriction was adopted as regards language or year of publication. All the articles found were published from 2006 to 2014.

### Search strategy

The research was conducted between August and November 2018, by two different reviewers (AVSSC, GAFJ) in the following databases: PubMed, Medline, Lilacs, Scielo and Cochrane.

The search strategy used corresponded to the following descriptors: “bariatric surgery” AND “dental erosion” OR “bariatric surgery” AND “dental erosion” AND “gastroesophageal reflux disease”.

### Tracing methods 

**FIGURE 1:**
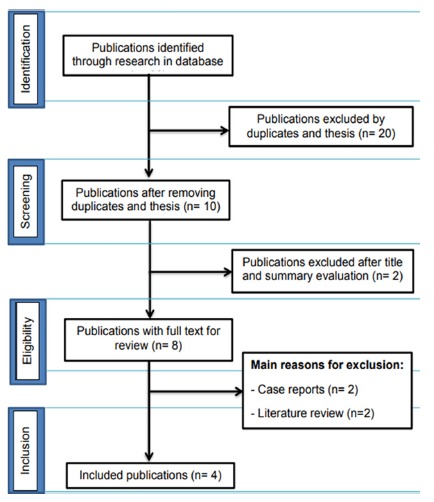
PRISMA flow diagram of four stages for collection of the study data, showing the number of studies identified, selected, eligible and included in the systematic review.

The electronic search recovered 30 abstracts and titles (Figure 1). After removing the duplicated references, a total of eight abstracts and titles were read and analyzed by two independent, calibrated reviewers

Two reviewers conducted the primary research by titles and abstracts. The same researchers evaluated the full manuscript, observing whether or not they met the inclusion/exclusion criteria, or those that had insufficient data in the title and abstract. Any disagreement was resolved by discussion between the reviewers. Disagreements were resolved by consensus between the reviewers, and also by a 3^rd^ reviewer, who was the supervisor responsible for the present research (SHCSP). When the results of a study were published more than once, or presented in various publications by one and the same author, they were included in the review once only.

The primary authors of the respective studies were contacted to request additional data in the event that there were relevant data that were absent or unavailable.

### Types of results

The following data of the respective articles were extracted and duly tabulated: authors, year of publication, country where the study was conducted, study design, place where the exam was conducted, sample size, participants’ age, measures of tooth wear index, statistical analysis used for tooth wear, and results with reference to tooth wear observed.

## RESULTS

A flow diagram that describes the search strategy and process for selection of the articles at each stage of this systematic review is provided in Figure 1. Thirty articles were identified in the electronic search. Twenty-two of the articles were discarded after analysis of the titles and abstracts, resulting in eight studies that were submitted to full text analysis. After evaluating the 8 complete texts, the literature review article and case studies were excluded. At the end, four articles remained, on which this review was based (Table 1).

**TABLE 1:** Cross-sectional and longitudinal studies included in this systematic review

Author (Year)	Country	Study design	Local of study	Sample size	Age	Dental wear measurement	Statistical analysis	Results
Moura-Grec (2014)	Brazil	Longitudinal (Cohort)	Hospital	GE:59 GC:51	GE:38.41±10.98 GC:41.68±10.84	Dental Wear Index (DWI)	t-Test	DWI (enamel): BBS* Mean: 19.80 ± 9.23 ABS* 17.01 ± 8.65 DWI (dentin): BBS* 9.89 ± 9.18 ABS* 15.68 ± 10.76 Eutrophic Group Enamel 16.7 % Dentin 11.9% BBS* Enamel 19.84% Dentin 10.59% ABS* (6 months) Enamel 16.19% Dentin 16.67% ABS* (12 months) Enamel 16.36% Dentin 17.53%
Marsicano (2011)	Brazil	Longitudinal	Hospital	54 patients	GE and GC:40.5±9.7	Dental Wear Index (DWI)	ANOVA	DWI (enamel): BBS* N=8 (14.8%) ABS* (3 months) N=3 (12.5%) ABS* (6 months) N=0 (0%) DWI (dentin): BBS* N=46 (85.2%) ABS* (3 months) N=21 (87.5%) ABS* (6 months) N=14 (100%) 25.4±9.3 pre-operative 27.4 ±12.7 (3 months) 32.7 ±10.2 (6 months)
Marsicano (2012)	Brazil	Cross-sectional	Hospital	GE 52 GC 50	GE: 39.6 ± 9.6 GC: 35.55 ±10.2	Dental Wear Index (DWI)	t-Test	DWI (enamel): Control Group N=12 (24%) Bariatric Group N=11 (21.15%) **DWI (dentin):** Control Group N=38 (76%) Bariatric Group N=40 (76.93%)
Heling (2006)	Jerusalem	Cross-sectional	Hospital	113	40 ± 10.24	Self-reported dental hypersensitivity	Chi-square Test	37% reported greater dentin hypersensitivity after surgery

*BBS=before bariatric surgery; *ABS=after bariatric surgery

All the studies included were published between 2006 and 2014. Table 1 summarizes the characteristics of each of the four studies included, highlighting the main result of each article relative to tooth wear. A total of 379 patients were analyzed in this systematic review. The majority of them were women. The mean of age was 40.5±9.7 years. 

## DISCUSSION

The evidences found in this systematic review indicated that bariatric surgery could improve the systemic condition of the patients; however, there was a negative impact on oral health, particularly with reference to increase in the occurrence of tooth wear.

Association between tooth wear and bariatric surgery was investigated in four studies^4^ ^,^ ^11^ ^,^ ^12^ ^,^ ^16^. Among them, three articles analyzed tooth wear according to the Dental Wear Index (DWI)^11^ ^,^ ^12^ ^,^ ^16^. On the other hand, the fourth article used a standardized self-reported questionnaire with regard to the presence of tooth wear in patients, according to dentin hypersensitivity^4^. The DWI is a standardized index, well established in the literature, which guaranteed high reliability of the data obtained in epidemiological researches^2^ ^,^ ^21^.

The studies demonstrated that the majority of patients who underwent bariatric surgery presented frequent vomiting, characterized by the presence of gastroesophageal reflux. Important to emphasize is that the vomiting generated high levels of acid in the mouth and that other gastric disturbances were important risk factors for the occurrence of tooth wear^10^ ^,^ ^11^ ^,^ ^24^.

In the longitudinal study of Moura Grec et al.^16^, 30% of the tooth surfaces presented tooth wear before the bariatric surgery, of which 20% were restricted to enamel and 10% in dentin. Six months after bariatric surgery, there was a 6% increase in wear in dentin. In this aforementioned study, the authors pointed out the increase in anxiety as one of the factors that contributed to the increase in severity of tooth wear after bariatric surgery. 

In 2011, in a longitudinal study Marsicano et al.^11^ emphasized that all the patients evaluated before the surgical intervention presented a high level of tooth wear, with 14.8% of the patients presenting tooth wear restricted to enamel, and 85.2% in dentin. Three months after bariatric surgery, 12.5% and 87.5% of the patients presented wear in enamel and dentin, respectively. And six months after bariatric surgery, 100% of the patients presented tooth wear involving dentin. 

In view of the findings presented, the need for dental attention in both the pre- and post-surgery periods was observed, considering the increase in severity of non-carious lesions. 

In a cross-sectional study Marsicano et al.^12^ showed that only one patient evaluated after bariatric surgery presented no clinical sign of tooth wear. On the other hand, 21.15% of the patients presented wear in enamel, and 76.93%, in dentin. The increase in prevalence and severity of this condition occurred due to the episodes of chronic vomiting and changed dietary pattern after bariatric surgery, because the patients began to ingest foods more frequently, although in smaller quantities. 

Heling et al.^4^ verified the presence of tooth wear after bariatric surgery, by means of a questionnaire related to the presence of dentin sensitivity. The limitation of this study was that no standardized index was used to qualify or quantify tooth wear itself. The authors pointed out that 37% of the patients presented a higher level of dentin sensitivity after bariatric surgery. This fact, which could be related to the possible occurrence of lesions in dentin, was stated according to the patient’s perception. 

Another point to consider refers to the type of surgery used in the treatment of obesity. In three studies^11^ ^,^ ^12^ ^,^ ^16^, the type was the Roux-en-Y gastric bypass, bariatric surgery with both restrictive and malabsorptive features. After operation, patients may present with frequent episodes of vomiting, which is a risk commonly associated with this type of surgery. These frequent episodes of vomiting may contribute to the increase in prevalence and severity of tooth wear, resulting from acid challenge associated with the effects of mechanical abrasion.

Others studies that have been conducted in different contexts and were not included in this review, have shown similar findings related to the possible association between tooth wear, gastroesophageal reflux and bariatric surgery. It is important to highlight that only the longitudinal and cross-sectional studies were included in this systematic review. Nevertheless, it must be considered that the cohort studies included in this review provided greater force of association for confirming this scientific evidence^14^ ^,^ ^18^.

The consistency related to possible causal association between tooth wear and bariatric surgery was strong. There were four studies analyzing this question, and all of them demonstrated this association. Future studies must be conducted to enable these evidences to be more clearly elucidated.

As limitations of the present systematic review, the low number of studies on the topic may be cited, since the longitudinal studies had short periods of follow-up, and there were no randomized clinical trials. Studies with follow-up periods longer than two years would improve the scientific evidence with regard to cause-effect of the studied outcomes. Therefore, the findings of this study must be extrapolated with caution. Moreover, half of the studies included in this review had a cross-sectional design, making it impossible the causal and temporal inferences about the association between tooth wear, gastroesophageal reflux and bariatric surgery. However, the force of association for inferring causality was based on the longitudinal studies included. Another question to be suggested refers to the association between the three outcomes presented in the cohort studies that used convenience samples, pointing out a gap in the literature. As a strong point, the authors emphasize that the systematic review elucidated the relations between the three outcomes analyzed, pointing out the importance to both clinical dentists and public health policy formulators, when considering the oral, systemic and surgical conditions of the individual in health management.

## CONCLUSION

The results of this systematic review showed association between tooth wear, gastroesophageal reflux and bariatric surgery. However, longitudinal studies of long duration must be conducted to test the causal and temporal relations between the three outcomes analyzed.
